# Change in the Estimated Glomerular Filtration Rate Over Time and Risk of First Stroke in Hypertensive Patients

**DOI:** 10.2188/jea.JE20210242

**Published:** 2023-03-05

**Authors:** Panpan He, Huan Li, Zhuxian Zhang, Yuanyuan Zhang, Tengfei Lin, Yun Song, Lishun Liu, Min Liang, Jing Nie, Binyan Wang, Yong Huo, Fan Fan Hou, Xiping Xu, Xianhui Qin

**Affiliations:** 1 Division of Nephrology, Nanfang Hospital, Southern Medical University; National Clinical Research Center for Kidney Disease; State Key Laboratory of Organ Failure Research; Guangdong Provincial Institute of Nephrology, Guangdong Provincial Key Laboratory of Renal Failure Research, Guangzhou Regenerative Medicine and Health Guangdong Laboratory, Guangzhou, China; 2 Beijing Advanced Innovation Center for Food Nutrition and Human Health, College of Food Science and Nutritional Engineering, China Agricultural University, Beijing, China; 3 Institute of Biomedicine, Anhui Medical University, Hefei, China; 4 Shenzhen Evergreen Medical Institute, Shenzhen, China; 5 Department of Cardiology, Peking University First Hospital, Beijing, China

**Keywords:** change in eGFR, first stroke, first ischemic stroke, hypertension

## Abstract

**Background:**

The association between changes in estimated glomerular filtration rate (eGFR) over time and the risk of stroke remains inconclusive. We aimed to evaluate the relation of eGFR change during the China Stroke Primary Prevention Trial (CSPPT) with the risk of first stroke during the subsequent post-trial follow-up.

**Methods:**

A total of 11,742 hypertensive participants with two eGFR measurements (median measure interval, 4.4; interquartile range, 4.2–4.6 years) and without a history of stroke from the CSPPT were included in this analysis.

**Results:**

Over a median post-trial follow-up of 4.4 years, 729 first strokes were identified, of which 635 were ischemic, 88 were hemorrhagic, and 6 were uncertain types of strokes. Compared with those with 1 to <2% per year increase in eGFR (with the lowest stroke risk), those with an increase in eGFR of ≥4% per year had significantly increased risks of first stroke (adjusted hazard ratio [HR] 1.96; 95% confidence interval [CI], 1.10–3.50) and first ischemic stroke (adjusted HR 2.14; 95% CI, 1.17–3.90). Similarly, those with a decline in eGFR of ≥5% per year also had significantly increased first stroke (adjusted HR 2.13; 95% CI, 1.37–3.31) and first ischemic stroke (adjusted HR 1.89; 95% CI, 1.19–3.02) risk. However, there was no significant association between eGFR change and first hemorrhagic stroke. A similar result was found when the change in eGFR was quantified as an absolute annual change.

**Conclusion:**

In Chinese hypertensive patients, both the decline and increase of eGFR levels were independently associated with the risks of first stroke or first ischemic stroke.

## INTRODUCTION

Stroke is the leading cause of mortality and disability in China and the second leading cause of death in the world.^1^^,^^2^ Hypertension is one of the most important risk factors for stroke.^3^^,^^4^ As the traditional risk factors cannot account for all stroke risk, it is of great clinical importance to identify more modifiable risk factors to improve primary prevention of stroke and reduce the related severe disease burden, especially in hypertensive patients.

Chronic kidney disease (CKD) is a worldwide public health problem, with increasing prevalence, poor outcomes, and high treatment costs.^5^ Previous studies have demonstrated an association between estimated glomerular filtration rate (eGFR) and stroke risk.^6^ However, most of the studies predominantly considered eGFR at one point in time (the baseline), without the consideration of how the change in eGFR over time influences the risk of stroke. Indeed, there has been a growing interest in the association between change in eGFR and risk of end-stage renal disease (ESRD),^7^ cardiovascular disease (CVD),^8^ and mortality.^9^ However, only a few of them have reported the association between the changes in eGFR over time with the risk of stroke, and they reported inconsistent findings.^8^^,^^10^^–^^15^ Moreover, although a Japanese longitudinal 10-year follow-up study^16^ reported that the rate of eGFR decline was significantly higher among participants with hypertension compared to the general population, few related studies have been conducted in hypertensive patients. More importantly, the possible effect modifiers on the association between eGFR change and first stroke have not been comprehensively evaluated in previous studies.

Using data from the China Stroke Primary Prevention Trial (CSPPT) and its post-trial follow-up, we aimed to investigate the association between change in eGFR over time and the risk of first stroke in hypertensive adults. We explored change in eGFR using two indices: the annual percentage change and the absolute annual change.

## METHODS

### Study design and population

The study design and major results of the CSPPT^17^^–^^20^ and the renal sub-study of the CSPPT^21^ have been reported previously. Briefly, the CSPPT was a randomized, double-blind, controlled trial conducted from May 19, 2008, to August 24, 2013, in 32 communities in China. Eligible participants were men and women aged 45 to 75 years who had hypertension, defined as seated, resting systolic blood pressure ≥140 mm Hg or diastolic blood pressure ≥90 mm Hg at both the screening and recruitment visit, or who were on antihypertensive medication. The major exclusion criteria included a history of physician-diagnosed stroke, heart failure, myocardial infarction, coronary revascularization, or congenital heart disease. The renal sub-study of the CSPPT (*n* = 15,104) enrolled eligible CSPPT participants from 20 communities in Jiangsu Province, excluding those with an eGFR <30 mL/min/1.73 m^2^ or missing eGFR at baseline.

The post-trial follow-up of the CSPPT^22^ was conducted from August 24, 2013, to December 31, 2017, in Lianyungang of Jiangsu Province.

The first collection of overnight fasting venous blood samples and spot urine samples of each study participant was carried out at the baseline of CSPPT in 2008, while the second collection of samples was at the exit visit of CSPPT in 2013. The median measurement interval was 4.4 (interquartile range [IQR], 4.2–4.6) years. The outcome ascertainment was performed during the post-trial follow-up (From August 24, 2013, to December 31, 2017).

Overall, of the 15,104 enrolled participants of the renal sub-study, the current study included 12,916 participants who completed the exit site visit of CSPPT and had two eGFR measurements. After further excluding subjects with a history of stroke at the exit site visit of CSPPT (*n* = 345), and without endpoint information (*n* = 829), a total of 11,742 hypertensive subjects were included in the final analysis (eFigure 1).

The CSPPT (Clinical Trials.gov, NCT00794885) was approved by the Ethics Committee of the Institute of Biomedicine, Anhui Medical University, Hefei, China (FWA assurance number: FWA00001263). The current study was approved by the Ethics Committee of the First People’s Hospital of Lianyungang, Lianyungang, China, and the Ethics Committee of Nanfang Hospital, Guangzhou, China. Informed consent was waived for the retrospective nature and the lack of participant interaction. The data that support the findings of this study will be available from the corresponding authors upon request, after the request is submitted and formally reviewed and approved by the Ethics Committee of the Institute of Biomedicine, Anhui Medical University; the Ethics Committee of the First People’s Hospital of Lianyungang; and the Ethics Committee of Nanfang Hospital.

### Laboratory assays

Serum creatinine, total homocysteine (tHcy), uric acid, fasting lipids, and fasting glucose were measured using automatic clinical analyzers (Beckman Coulter, Brea, CA, USA) at the core laboratory of the National Clinical Research Center for Kidney Disease, Guangzhou, China. Serum folate and vitamin B12 were measured by a commercial laboratory using a chemiluminescent immunoassay (New Industrial, Shenzhen, China). Specifically, serum creatinine was measured using an enzymatic assay that has been calibrated to be isotope dilution mass spectrometry traceable. The coefficient of variation for the assay was 1.4%. Proteinuria was determined using a dipstick test (Dirui-H100; Jilin, China).

Diabetes was defined as a fasting glucose ≥7.0 mmol/L or, use of glucose-lowering drugs or, self-reported history of diabetes. Participants with an eGFR less than 60 mL/min/1.73 m^2^ and/or proteinuria at baseline were classified as having CKD. Hyperfiltration was defined as an eGFR greater than 95th percentile, and a normal glomerular filtration rate was defined as an eGFR between the 25th and 75th percentiles after stratification for age decade and sex.^23^^,^^24^

### Change in eGFR

eGFR was calculated using the Chronic Kidney Disease Epidemiology Collaboration (CKD-EPI) equation.^25^ Change in eGFR over time was estimated using the two eGFR measurements for each patient at the baseline and exit visit of CSPPT, with a median measurement interval of 4.4 (IQR, 4.2–4.6) years.

The primary method we used to describe the change in eGFR was the annual percentage change, which was calculated as ([eGFR at baseline/eGFR at exist visit]^^^[1/years elapsed between visits] − 1)^*^100. The annual percentage change in eGFR was categorized as ≤−5%, −5% to <−4%, −4% to <−3%, −3% to <−2%, −2% to <−1%, −1% to <1%, 1% to <2%, 2% to <3%, 3% to <4%, 4% to <5%, and ≥5% per year.

In the sensitivity analysis, we used the absolute annual change as an alternate way to define the change in eGFR over time. The absolute annual change was calculated as follow: (eGFR at exist visit − eGFR at baseline)/(years elapsed between visits). The absolute annual change in eGFR was categorized as ≤−5, −5 to <−4, −4 to <−3, −3 to <−2, −2 to <−1, −1 to <1, 1 to <2, 2 to <3, 3 to <4, 4 to <5, and ≥5 mL/min per 1.73 m^2^ per year.

### Outcomes

The primary outcome of this study was the first stroke. Secondary outcomes include first ischemic stroke and first hemorrhagic stroke. According to the International Classification of Diseases, 10th Revision (ICD-10), the study outcome (first nonfatal or fatal stroke) included first ischemic stroke (I63), first hemorrhagic stroke (I60–I61) and no type stroke (I64).

Information on stroke incidence was obtained via the Center for Disease Control and Prevention of Ganyu and Donghai counties and checked against the national health insurance system with electronic linkage to all hospitalizations or ascertained through active follow-up. As shown in the government documentation,^26^^,^^27^ local authorities from medical institutions are required to report all new cases of stroke to the local Center for Disease Control and Prevention. They are required to submit a report card, which includes information on demographics, diagnostic basis, and date of stroke on the 28th of each month. Trained officials and the local Center for Disease Control and Prevention carry out the quality control, including finding and deleting repeated cases, error checking, and determining any missed cases. Besides, 5% of all uploaded cases are randomly chosen for further confirmation by phone or door-to-door interviews.

### Statistical analysis

Baseline characteristics (at the first measurement) are presented as means and standard deviations (SD) for continuous variables and proportions for categorical variables by the annual percentage change in eGFR (≤−5%, −5% to <1%, 1% to <2%, 2% to <4%, or ≥4% per year). The differences in population characteristics were compared using ANOVA tests or chi-square tests, accordingly.

Cox proportional hazards models were used to estimate the hazard ratios (HRs) and 95% confidence intervals (CIs) for the risk of first stroke and its subtypes associated with each group of change in eGFR, with and without adjustment for age, sex, treatment group, body mass index (BMI), systolic blood pressure (SBP), fasting glucose, total cholesterol (TC), triglycerides, high-density lipoprotein cholesterol (HDL-C), tHcy, smoking and drinking statuses, proteinuria, eGFR at baseline (the first measurement), as well as time-averaged SBP during treatment.

In addition, possible modifications of the association between annual percentage change in eGFR (≤−5%, −5% to <1%, 1% to <2%, 2% to <4%, or ≥4% per year) and first stroke was also assessed for the following variables: sex, age (<60 vs ≥60 years), BMI (<24 vs ≥24 kg/m^2^), current smoking and alcohol drinking status (yes vs no), SBP (<160 vs ≥160 mm Hg), tHcy (<15 vs ≥15 µmol/L), TC (<5.2 vs ≥5.2 mmol/L), diabetes (yes vs no), CKD (yes vs no), treatment group (enalapril vs enalapril-folic acid) at baseline (the first measurement), and time-averaged SBP during the treatment period (<140 vs ≥140 mm Hg).

A two-tailed *P* < 0.05 was considered statistically significant in all analyses. R software (version 3.5.0; R Foundation for Statistical Computing, Vienna, Austria) was used for all statistical analyses.

## RESULTS

### Baseline characteristics of participants

The flow chat of participants was presented in eFigure 1. A total of 11,742 participants were included in the final analysis.

The average age of the participants was 59.4 (SD, 7.4) years old and 4,424 (37.7%) were men. The distribution of annual percentage change appeared normal and centered near the origin. The mean annual percentage change in eGFR was −1.34% (SD, 3.35), with a median of −1.02% (IQR, −2.39 to 0.05).

Table 1 shows the baseline characteristics (at the first measurement) of participants according to the annual percentage change in eGFR. Participants experiencing a greater annual percentage decline or increase in eGFR were older, more likely to be female, had higher triglycerides, fasting glucose, creatinine levels, and a higher prevalence of proteinuria at baseline (Table 1).

**Table 1.** Baseline characteristics (at the first measurement) of study participants by annual percentage change in eGFR categories^a^

Characteristics	Annual percentage change in eGFR (% per year)					*P* value

	≤−5	−5 to <1	1 to <2	2 to <4	≥4	
*N*	1,030	9,137	645	609	321	
Age, years	62.4 (7.7)	59.1 (7.3)	58.7 (7.3)	59.5 (7.5)	59.2 (7.9)	<0.001
Male, *n* (%)	374 (36.3)	3,409 (37.3)	269 (41.7)	253 (41.5)	119 (37.1)	0.045
BMI, kg/m^2^	25.8 (3.8)	25.6 (3.5)	26.0 (3.5)	25.9 (3.5)	25.6 (3.3)	0.076
BP, mm Hg						
Baseline SBP	174.0 (22.7)	167.9 (20.3)	165.7 (19.9)	166.2 (21.1)	164.8 (22.6)	<0.001
Baseline DBP	95.5 (12.5)	95.2 (11.7)	95.3 (11.7)	95.7 (12.1)	94.7 (13.0)	0.690
Time-averaged SBP during treatment	141.6 (11.7)	139.0 (10.6)	139.0 (10.4)	139.7 (11.6)	138.9 (11.3)	<0.001
Time-averaged DBP during treatment	83.0 (7.7)	83.4 (7.2)	84.0 (7.0)	84.4 (7.2)	83.8 (7.7)	<0.001
Current smoking, *n* (%)	228 (22.1)	1,967 (21.5)	136 (21.1)	131 (21.5)	64 (19.9)	0.283
Current alcohol drinking, *n* (%)	211 (20.5)	2,022 (22.1)	151 (23.4)	140 (23.0)	68 (21.2)	0.717
Enalapril–folic acid, *n* (%)	486 (47.2)	4,542 (49.7)	343 (53.2)	316 (51.9)	171 (53.3)	0.079
Hyperfiltration, *n* (%)	62 (6.0)	514 (5.6)	7 (1.1)	5 (0.8)	1 (0.3)	<0.001
CKD, *n* (%)	198 (19.8)	783 (8.9)	63 (10.2)	80 (13.7)	108 (35.3)	<0.001
**Laboratory tests**						
Total cholesterol, mmol/L	5.6 (1.2)	5.6 (1.1)	5.9 (1.2)	6.1 (1.3)	6.5 (1.9)	<0.001
Triglycerides, mmol/L	1.8 (1.0)	1.7 (1.4)	1.6 (0.9)	1.6 (0.8)	1.7 (1.2)	0.011
HDL-C, mmol/L	1.3 (0.4)	1.3 (0.4)	1.3 (0.4)	1.3 (0.3)	1.3 (0.4)	0.006
Fasting glucose, mmol/L	6.2 (2.3)	6.0 (1.7)	6.0 (1.3)	6.0 (1.4)	6.8 (2.1)	<0.001
Folate, ng/mL	7.5 (3.4)	7.7 (3.2)	7.7 (3.4)	7.9 (3.0)	7.9 (3.3)	0.258
Total homocysteine, µmol/L	15.3 (8.9)	14.2 (8.7)	15.4 (8.8)	15.8 (10.1)	18.2 (11.6)	<0.001
Creatinine, µmol/L	67.0 (18.3)	62.3 (12.9)	71.9 (13.2)	76.8 (14.5)	89.9 (22.4)	<0.001
Proteinuria, *n* (%)	180 (18.1)	747 (8.5)	58 (9.4)	61 (10.4)	41 (13.6)	<0.001
**eGFR, mL/min/1.73 m^2^**						
At baseline	90.7 (15.5)	96.7 (10.7)	88.8 (11.2)	83.1 (12.0)	70.5 (15.6)	<0.001
At exist visit	63.1 (14.7)	91.3 (11.4)	94.7 (11.9)	93.8 (13.6)	94.7 (15.9)	<0.001

BMI, body mass index; BP, blood pressure; CKD, chronic kidney disease, DBP, diastolic blood pressure; eGFR, estimated glomerular filtration rate; HDL-C, high-density lipoprotein cholesterol; SBP, systolic blood pressure.

Variables are presented as mean (standard deviation) unless otherwise noted.

### Association between change in eGFR and the risk of study outcomes

Over a median post-trial follow-up of 4.4 years, 729 first-time strokes were identified, of which 635 were ischemic stroke, 88 were hemorrhagic stroke, and 6 were uncertain types of strokes.

**Figure 1.**
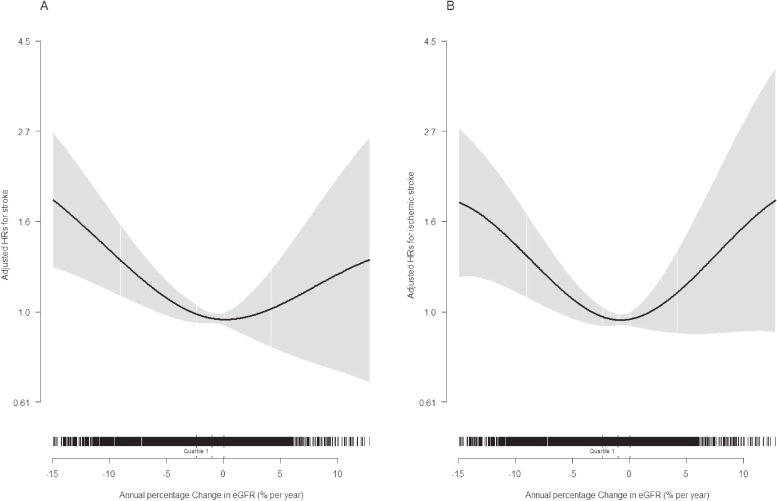
The relation between annual percentage change in eGFR and the study outcomes: first stroke (A); first ischemic stroke (B) in hypertensive patients. Adjusted for age, sex, treatment group, body mass index, systolic blood pressure (SBP), smoking and drinking status, fasting glucose, total cholesterol, triglycerides, high-density lipoprotein cholesterol, total homocysteine, proteinuria, estimated glomerular filtration rate (eGFR) at baseline (the first measurement), as well as time-averaged SBP during treatment.

Overall, we observed a U-shaped relation between percentage change in eGFR per year and first stroke (Figure 1A). Participants with an increase in eGFR of 1% to <2% per year had the lowest stroke risk. Accordingly, compared with those with 1% to <2% per year increase in eGFR, the adjusted HRs for participants with an increase in eGFR of ≥4% per year and a decline in eGFR of ≥5% per year were 1.96 (95% CI, 1.10–3.50) and 2.13 (95% CI, 1.37–3.31), respectively (Table 2). In our study, there were positive but no significant relations of hyperfiltration and CKD status at baseline with first stroke (eTable 1). Furthermore, the adjustments for hyperfiltration and CKD status did not substantially change the findings (eTable 2).

**Table 2.** The association between annual percentage change in eGFR and first stroke

Annual percentage change in eGFR (% per year)	*N*	Events (%)	Crude Models		Adjusted Models^a^	
	
			HR (95% CI)	*P* value	HR (95% CI)	*P* value
Categories						
≤−5	1,030	106 (10.3)	2.55 (1.67–3.89)	<0.001	2.12 (1.36–3.30)	<0.001
−5 to <−4	478	33 (6.9)	1.67 (1.00–2.77)	0.049	1.56 (0.93–2.64)	0.094
−4 to <−3	748	39 (5.2)	1.25 (0.77–2.04)	0.372	1.18 (0.71–1.97)	0.515
−3 to <−2	1,254	72 (5.7)	1.39 (0.89–2.16)	0.146	1.30 (0.81–2.07)	0.274
−2 to <−1	2,417	144 (6.0)	1.44 (0.95–2.17)	0.082	1.49 (0.97–2.29)	0.071
−1 to <1	4,240	249 (5.9)	1.41 (0.95–2.10)	0.087	1.52 (1.00–2.30)	0.049
1 to <2	645	27 (4.2)	Ref		Ref	
2 to <3	403	22 (5.5)	1.32 (0.75–2.31)	0.339	1.36 (0.77–2.42)	0.292
3 to <4	206	11 (5.3)	1.28 (0.64–2.59)	0.486	1.09 (0.51–2.35)	0.816
4 to <5	114	10 (8.8)	2.14 (1.04–4.42)	0.040	2.01 (0.90–4.49)	0.088
≥5	207	16 (7.7)	1.87 (1.01–3.47)	0.048	1.93 (1.01–3.72)	0.048
Categories						
≤−5	1,030	106 (10.3)	2.55 (1.67–3.89)	<0.001	2.13 (1.37–3.31)	<0.001
−5 to <1	9,137	537 (5.9)	1.42 (0.96–2.09)	0.077	1.45 (0.97–2.18)	0.072
1 to <2	645	27 (4.2)	Ref		Ref	
2 to <4	609	33 (5.4)	1.30 (0.78–2.17)	0.305	1.27 (0.75–2.16)	0.372
≥4	321	26 (8.1)	1.96 (1.15–3.37)	0.014	1.96 (1.10–3.50)	0.023

CI, confidence interval; HR, hazard ratio.

^a^Adjusted for age, sex, treatment group, body mass index, systolic blood pressure (SBP), smoking and drinking status, fasting glucose, total cholesterol, triglycerides, high-density lipoprotein cholesterol, total homocysteine, proteinuria, estimated glomerular filtration rate (eGFR) at baseline (the first measurement), as well as time-averaged SBP during treatment.

Consistently, a significantly higher risk of first ischemic stroke was observed in participants with an increase in eGFR of ≥4% per year (HR 2.14; 95% CI, 1.17–3.90) and with a decline in eGFR of ≥5% per year (HR 1.89; 95% CI, 1.19–3.02), compared with those with 1% to <2% per year increase in eGFR (Figure 1B and Table 3). However, there was no significant association between change in eGFR and first hemorrhagic stroke: compared with those with 1% to <2% per year increase in eGFR, the adjusted HRs of first hemorrhagic stroke for participants with an increase in eGFR of ≥4% per year and with a decline in eGFR of ≥5% per year were 0.78 (95% CI, 0.07–9.02) and 3.88 (95% CI, 0.88–17.15), respectively (Table 3).

**Table 3.** The association between annual percentage change in eGFR and ischemic or hemorrhagic stroke

Annual percentage change in eGFR (% per year)	*N*	Events (%)	Crude Models		Adjusted Models^a^	
			HR (95% CI)	*P* value	HR (95% CI)	*P* value
**Ischemic stroke**						
Categories						
≤−5	1,030	88 (8.5)	2.26 (1.45–3.53)	<0.001	1.89 (1.19–3.02)	0.007
−5 to <1	9,137	467 (5.1)	1.33 (0.89–1.98)	0.169	1.35 (0.88–2.06)	0.166
1 to <2	645	25 (3.9)	Ref			
2 to <4	609	30 (4.9)	1.28 (0.75–2.17)	0.364	1.32 (0.76–2.29)	0.321
≥4	321	25 (7.8)	2.04 (1.17–3.55)	0.012	2.14 (1.17–3.90)	0.013
**Hemorrhagic stroke**						
Categories						
≤−5	1,030	16 (1.6)	5.04 (1.16–21.91)	0.031	3.88 (0.88–17.15)	0.074
−5 to <1	9,137	66 (0.7)	2.33 (0.57–9.53)	0.238	2.30 (0.56–9.49)	0.251
1 to <2	645	2 (0.3)	Ref			
2 to <4	609	3 (0.5)	1.59 (0.27–9.54)	0.610	0.91 (0.13–6.46)	0.921
≥4	321	1 (0.3)	1.01 (0.09–11.13)	0.994	0.78 (0.07–9.02)	0.844

CI, confidence interval; HR, hazard ratio.

^a^Adjusted for age, sex, treatment group, body mass index, systolic blood pressure (SBP), smoking and drinking status, fasting glucose, total cholesterol, triglycerides, high-density lipoprotein cholesterol, total homocysteine, proteinuria, estimated glomerular filtration rate (eGFR) at baseline (the first measurement), as well as time-averaged SBP during treatment.

Similar results were obtained when the change in eGFR was defined as the absolute change in eGFR per year (eFigure 2).

### Stratified analyses by potential effect modifiers

**Figure 2.**
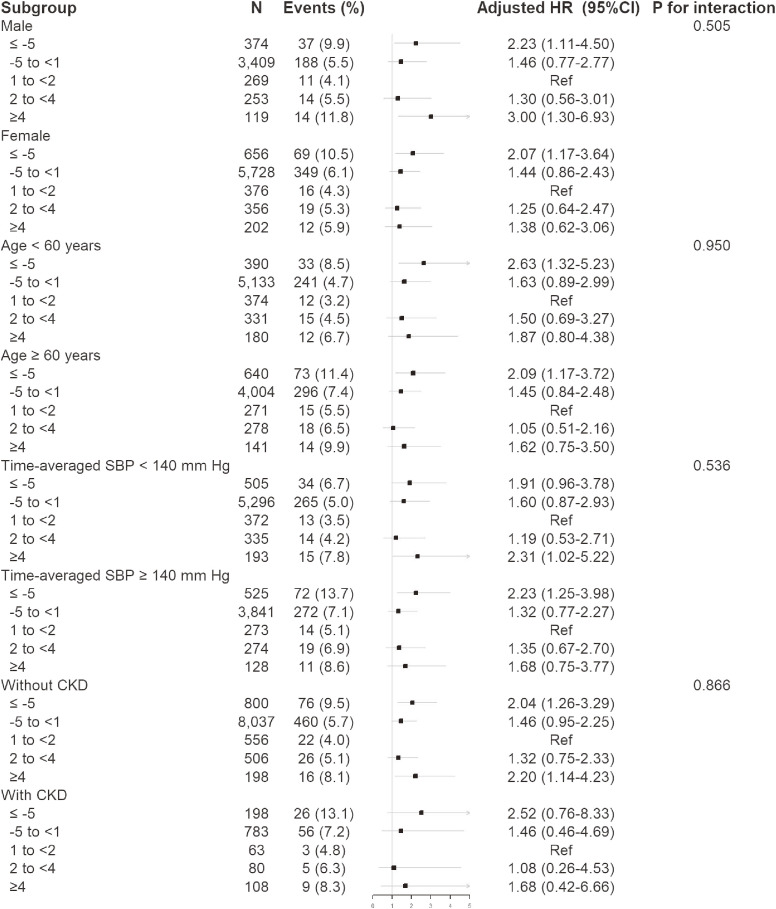
Stratified analyses by potential effect modifiers for first stroke. Adjusted for age, sex, treatment group, body mass index, systolic blood pressure (SBP), smoking and drinking status, fasting glucose, total cholesterol, triglycerides, high-density lipoprotein cholesterol, total homocysteine, proteinuria, estimated glomerular filtration rate (eGFR) at baseline (the first measurement), as well as time-averaged SBP during treatment, if not stratified. Boxes denote ORs, lines represent 95% CIs.

Stratified analyses were performed to assess the relation of the annual percentage change in eGFR (≤−5%, −5% to <1%, 1% to <2%, 2% to <4%, or ≥4% per year) and first stroke in various subgroups. None of the variables, including sex, age, BMI, treatment group, smoking and alcohol intake status, SBP, TC, tHcy, CKD and diabetes at baseline (the first measurement), as well as time-averaged SBP during the CSPPT treatment period, significantly modified the association between annual percentage change in eGFR and first stroke (*P* for all interactions >0.05) (Figure 2 and eTable 3).

Moreover, the sex-specific BMI status stratified analysis also showed that BMI did not significantly modify the association between annual percentage change in eGFR and first stroke in males or females (Males: *P* for interaction = 0.878; females: *P* for interaction = 0.669) (eTable 4).

## DISCUSSION

In this community-based hypertensive cohort, we found that change in eGFR over time was significantly associated with an increased risk of first stroke or first ischemic stroke. Compared with participants with 1% to <2% per year increase in eGFR, both declining and increasing eGFR were associated with a higher risk of first stroke or first ischemic stroke, independent of baseline hyperfiltration status, CKD status, and other covariates. Moreover, the results were consistent among participants with different baseline characteristics.

Actually, previous studies have linked the change in eGFR with stroke and reported inconsistent results. In community-based cohorts, Turin et al^8^ and Van Pottelbergh et al^11^ found that, compared to participants with stable eGFR, those with the greatest annual eGFR decline (≤−5 mL/min/1.73 m^2^ per year) had increased risk of stroke. Turin et al^13^ and Nagai et al^15^ also suggested that the decline in eGFR was a significant and independent risk factor for the incidence of stroke among the general population. Consistently, Guo et al^14^ reported an absolute decrease in eGFR ≥15 mL/min/1.73 m^2^ or a relative reduction (a decline of ≥25%) in eGFR independently predicted the risk for ischemic stroke at 6 months in patients with atrial fibrillation. Nevertheless, findings from the Cardiovascular Health Study (CHS),^10^ a community-based cohort of ambulatory elderly individuals, suggested that a rapid decline in eGFR (>3 mL/min per 1.73 m^2^ per year) was not significantly associated with stroke (HR 1.21; 95% CI, 0.95–1.53). Furthermore, a secondary analysis of the Antihypertensive and Lipid Lowering Treatment to Prevent Heart Attack Trial (ALLHAT)^12^ found that the annual decline in eGFR was not associated with stroke risk among hypertensive participants. It should be noted that most of this prior studies^8^^,^^10^^–^^13^ did not report any data on urinary protein, an important determinant of renal disease and cardiovascular outcomes^28^ and closely associated with the change in kidney function. Moreover, none of the studies thoroughly examined possible effect modifiers on the eGFR change-stroke association.

Our current study provided some new insights into this field. Overall, the change in eGFR over time was significantly associated with first stroke. First, consistent with previous studies,^8^^,^^11^^,^^13^^–^^15^ the risk of first stroke significantly increased with the decline in eGFR. This finding is biologically plausible based on the available literature, although the exact mechanisms remain to be delineated. Declining eGFR may contribute to increased stroke risk by aggravating endothelial dysfunction, inflammation,^29^ and oxidative stress,^30^ as well as through activation of the renin-angiotensin system induced by renal impairment.^31^ Furthermore, decreased kidney function may also be a biological integrating marker of adverse vascular conditions that cannot be fully captured by traditional risk factors.^32^

Second, our study expands the results of previously published studies by demonstrating that increasing eGFR over time was also associated with first stroke risk. Indeed, some reports have demonstrated that an increase in eGFR is associated with excess mortality in the general population.^9^ The mechanism underlying this association is still uncertain. Some researchers have mentioned that this phenomenon might be attributable to lower serum creatinine generation as a result of reduced muscle mass associated with chronic debilitating conditions.^33^ Nevertheless, our study showed that BMI levels did not significantly modify the association of eGFR with stroke risk.^34^ Furthermore, a rising eGFR could be seen with hyperfiltration in remnant nephrons, which could be associated with subsequent kidney disease progression and cardiovascular risk.^23^ However, the adjustments for hyperfiltration did not materially change our findings; therefore, hyperfiltration may possibly only partly affect the association. Overall, more studies are needed to verify our findings and further investigate the underlying mechanisms involved in this association.

In our study, the serum creatinine levels were measured in a single central laboratory and were, therefore, highly standardized. We also included a comprehensive adjustment and stratified analysis for a series of important confounders, especially those that may be associated with the change in kidney function, including BMI^35^ and proteinuria. However, our study also had some limitations. First, although a number of potential confounders had been adjusted in the analysis, it is possible that the results could be affected by unmeasured or unidentified factors. Second, our present study was conducted in Chinese hypertensive participants, so the generalizability of the results to the general Chinese population or other ethnicities should be further verified. Third, our study may be underpowered for evaluating the relation of eGFR change with the risk of hemorrhagic stroke. Fourth, only two eGFR measurements were available for each subject. As such, we could not evaluate the process of GFR change. More frequent measurements of GFR would have provided more information. Finally, we did not have accurate measurements for muscle mass. Therefore, our findings should be further confirmed in future studies.

### Conclusion

In this sample of Chinese hypertensive patients, we found that both declining and increasing eGFR over time were significantly associated with first stroke risk, independent of baseline hyperfiltration status, CKD status, and other covariates. If further confirmed, monitoring the trajectory of change in eGFR over time would be beneficial for clinical decision-making to identify patients at greater risk of stroke.

## SUPPLEMENTARY MATERIAL

eFigure 1. Flow chart of the participants

eFigure 2. The relation between absolute annual change in eGFR and the study outcomes: first stroke (A); first ischemic stroke (B) in hypertensive patients. Adjusted for age, sex, treatment group, body mass index, systolic blood pressure (SBP), smoking and drinking status, fasting glucose, total cholesterol, triglycerides, high-density lipoprotein cholesterol, total homocysteine, proteinuria, estimated glomerular filtration rate (eGFR) at baseline (the first measurement), as well as time-averaged SBP during treatment.

eTable 1. The relation between hyperfiltration status or chronic kidney disease at baseline and stroke^a^

eTable 2. The association between annual percentage change in eGFR and first stroke with further adjustments for hyperfiltration and chronic kidney disease status^a^

eTable 3. Stratified analyses by potential effect modifiers for first stroke^a^

eTable 4. Sex-specific BMI status stratified analysis^a^
