# Supplementary material for: Change in the Estimated Glomerular Filtration Rate Over Time and Risk of First Stroke in Hypertensive Patients
Source: J Epidemiol. 2023 Mar 5;33(3):142–9. doi: 10.2188/jea.JE20210242 (PMC9909174; doi:10.2188/jea.JE20210242)
Supplement: Supplementary file 1 [file je-33-142-s001.pdf]

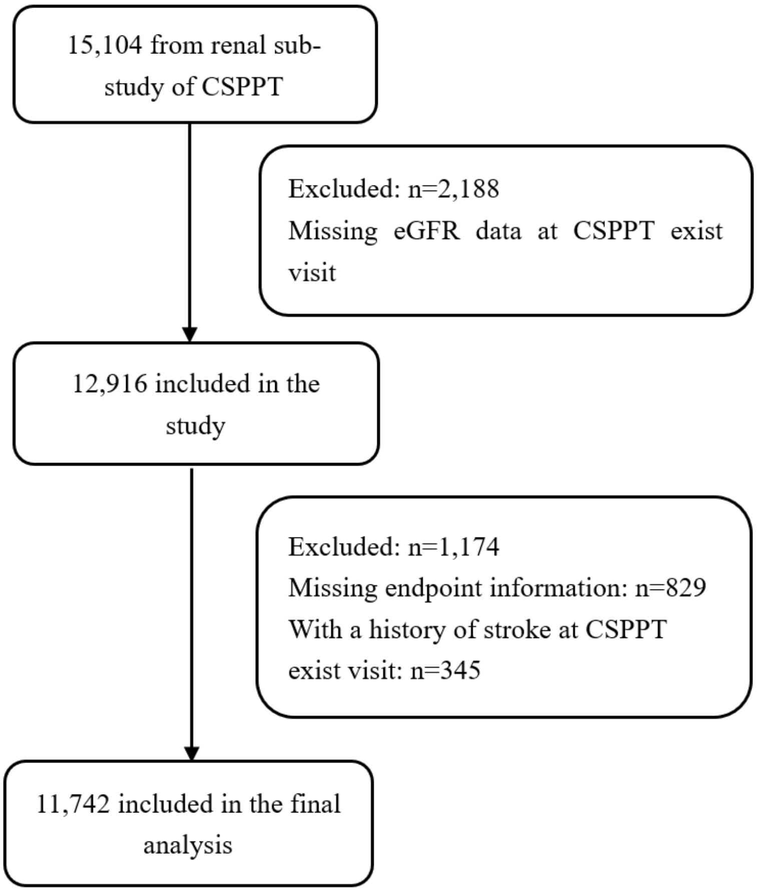

eFigure 1. Flow chart of the participants

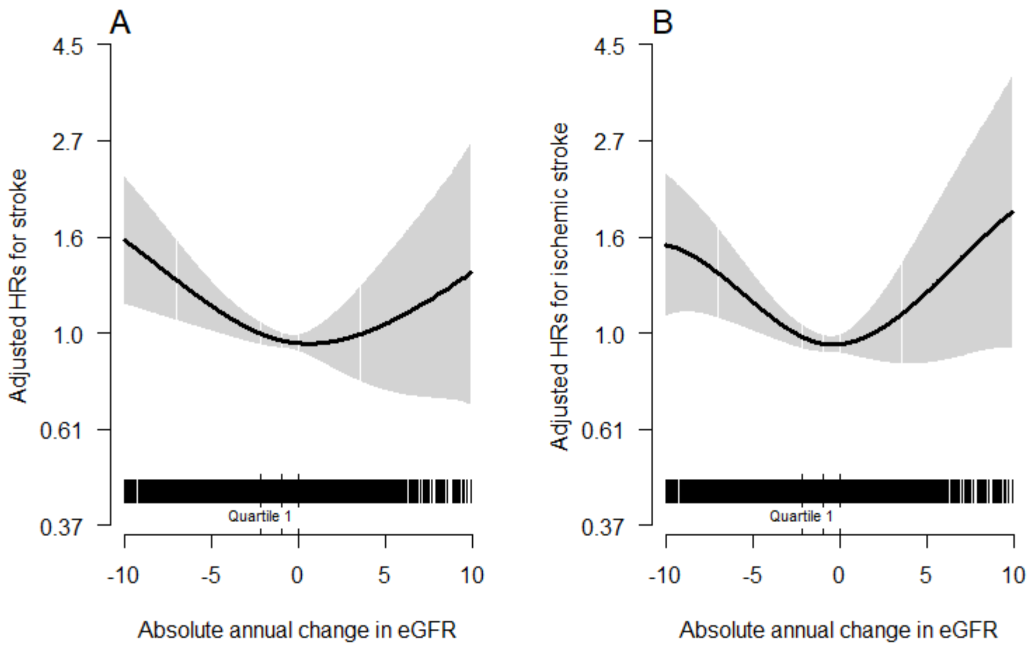

eFigure 2. The relation between absolute annual change in eGFR and the study outcomes: first stroke (A); first ischemic stroke (B) in hypertensive patients. Adjusted for age, sex, treatment group, body mass index, systolic blood pressure (SBP), smoking and drinking status, fasting glucose, total cholesterol, triglycerides, high-density lipoprotein cholesterol, total homocysteine, proteinuria, estimated glomerular filtration rate (eGFR) at baseline (the first measurement), as well as time-averaged SBP during treatment.

eTable 1. The relation between hyperfiltration status or chronic kidney disease at baseline and stroke[a]

	N	Events (%)	Crude Models		Adjusted Models	
			**HR (95% CI)**	***P* value**	**HR (95% CI)**	***P* value**
Filtration [a]						
Normal glomerular filtration	5,869	374 (6.4)	Ref		Ref	
Hyperfiltration	589	41 (7.0)	1.10 (0.79–1.51)	0.576	1.11 (0.79–1.56)	0.553
Chronic kidney disease [b]						
No	10,097	600 (5.9)	Ref		Ref	
Yes	1,232	99 (8.0)	1.37 (1.11–1.69)	0.004	1.15 (0.93–1.43)	0.207

CI, confidence interval; HR, hazard ratio.

[a] Adjusted for age, sex, treatment group, body mass index, systolic blood pressure (SBP), smoking and drinking status, fasting glucose, total cholesterol, triglycerides, high-density lipoprotein cholesterol, total homocysteine, proteinuria at baseline, as well as time-averaged SBP during treatment in the hyperfiltration models;

[b] Adjusted for age, sex, treatment group, body mass index, systolic blood pressure (SBP), smoking and drinking status, fasting glucose, total cholesterol, triglycerides, high-density lipoprotein cholesterol, total homocysteine at baseline, as well as time-averaged SBP during treatment in the chronic kidney disease models.

Hyperfiltration was defined as an eGFR greater than 95th percentile, and a normal glomerular filtration rate was defined as an eGFR between the 25th and 75th percentiles after stratification for age decade and sex.

eTable 2. The association between annual percentage change in eGFR and first stroke with further adjustments for hyperfiltration and chronic kidney disease status[a]

Annual percentage change in eGFR (percent/year)	N	Events (%)	Crude Models		Adjusted Models	
			HR (95% CI)	*P* value	HR (95% CI)	*P* value
Categories						
≤−5	1,030	106 (10.3)	2.55 (1.67–3.89)	<0.001	2.11 (1.35–3.28)	<0.001
−5 to <−4	478	33 (6.9)	1.67 (1.00–2.77)	0.049	1.55 (0.92–2.62)	0.099
−4 to <−3	748	39 (5.2)	1.25 (0.77–2.04)	0.372	1.17 (0.70–1.94)	0.552
−3 to <−2	1,254	72 (5.7)	1.39 (0.89–2.16)	0.146	1.27 (0.80–2.02)	0.306
−2 to <−1	2,417	144 (6.0)	1.44 (0.95–2.17)	0.082	1.48 (0.96–2.27)	0.073
−1 to <1	4,240	249 (5.9)	1.41 (0.95–2.10)	0.087	1.52 (1.00–2.29)	0.048
1 to <2	645	27 (4.2)	Ref		Ref	
2 to <3	403	22 (5.5)	1.32 (0.75–2.31)	0.339	1.36 (0.77–2.42)	0.291
3 to <4	206	11 (5.3)	1.28 (0.64–2.59)	0.486	1.09 (0.51–2.34)	0.825
4 to <5	114	10 (8.8)	2.14 (1.04–4.42)	0.040	1.98 (0.89–4.41)	0.093
≥5	207	16 (7.7)	1.87 (1.01–3.47)	0.048	1.85 (0.98–3.50)	0.058
Categories						
≤−5	1,030	106 (10.3)	2.55 (1.67–3.89)	<0.001	2.12 (1.36–3.30)	<0.001
−5 to <1	9,137	537 (5.9)	1.42 (0.96–2.09)	0.077	1.45 (0.97–2.16)	0.073
1 to <2	645	27 (4.2)	Ref			
2 to <4	609	33 (5.4)	1.30 (0.78–2.17)	0.305	1.27 (0.75–2.15)	0.375
≥4	321	26 (8.1)	1.96 (1.15–3.37)	0.014	1.89 (1.07–3.33)	0.028

CI, confidence interval; eGFR, estimated glomerular filtration rate; HR, hazard ratio.

[a] Adjusted for age, sex, treatment group, body mass index, systolic blood pressure (SBP), smoking and drinking status, fasting glucose, total cholesterol, triglycerides, high-density lipoprotein cholesterol, total homocysteine, hyperfiltration status, chronic kidney disease at baseline, as well as time-averaged SBP during treatment.

eTable 3. Stratified analyses by potential effect modifiers for first stroke[a]

Subgroup	N	Events (%)	Adjusted HR (95% CI)	N	Events (%)	Adjusted HR (95% CI)	*P* for interaction
**BMI, kg/m^2**		**<24**			**≥24**		**0.502**
≤ −5	345	28 (8.1)	1.33 (0.62–2.85)	684	78 (11.4)	2.61 (1.52–4.50)	
−5 to <1	3,033	176 (5.8)	1.20 (0.61–2.35)	6,103	361 (5.9)	1.58 (0.96–2.62)	
1 to <2	194	9 (4.6)	Ref	451	18 (4.0)	Ref	
2 to <4	182	10 (5.5)	1.04 (0.41–2.63)	427	23 (5.4)	1.39 (0.73–2.65)	
≥4	99	7 (7.1)	1.50 (0.55–4.09)	222	19 (8.6)	2.15 (1.07–4.33)	
**Total cholesterol, mmol/L**		**<5.2**			**≥5.2**		**0.197**
≤ −5	407	35 (8.6)	1.33 (0.63–2.79)	623	71 (11.4)	2.79 (1.61–4.82)	
−5 to <1	3,254	177 (5.4)	1.06 (0.54–2.08)	5,882	360 (6.1)	1.71 (1.03–2.84)	
1 to <2	178	9 (5.1)	Ref	467	18 (3.9)	Ref	
2 to <4	148	10 (6.8)	1.33 (0.54–3.28)	461	23 (5.0)	1.26 (0.65–2.41)	
≥4	75	9 (12.0)	2.02 (0.77–5.32)	246	17 (6.9)	1.90 (0.93–3.86)	
**Homocysteine, μmol/L**		**<15**			**≥15**		**0.666**
≤ −5	688	71 (10.3)	1.86 (1.11–3.11)	340	35 (10.3)	2.99 (1.26–7.13)	
−5 to <1	6,809	391 (5.7)	1.27 (0.80–2.03)	2,323	146 (6.3)	2.03 (0.89–4.61)	
1 to <2	434	19 (4.4)	Ref	211	8 (3.8)	Ref	
2 to <4	396	23 (5.8)	1.28 (0.69–2.36)	212	10 (4.7)	1.35 (0.48–3.79)	
≥4	161	10 (6.2)	1.52 (0.70–3.31)	159	15 (9.4)	2.99 (1.13–7.89)	
**Diabetes**		**No**			**Yes**		**0.776**
≤ −5	889	90 (10.1)	2.23 (1.36–3.64)	141	16 (11.3)	1.90 (0.69–5.20)	
−5 to <1	8,017	461 (5.8)	1.50 (0.95–2.35)	1,119	76 (6.8)	1.32 (0.53–3.28)	
1 to <2	543	22 (4.1)	Ref	102	5 (4.9)	Ref	
2 to <4	512	26 (5.1)	1.30 (0.72–2.34)	97	7 (7.2)	1.15 (0.35–3.79)	
≥4	229	21 (9.2)	2.37 (1.26–4.46)	92	5 (5.4)	1.05 (0.28–4.00)	
**Folic acid supplementation**		**No**			**Yes**		**0.809**
≤ −5	544	60 (11.0)	2.21 (1.18,4.12)	486	46 (9.5)	2.01 (1.08,3.75)	
−5 to <1	4595	263 (5.7)	1.37 (0.77,2.46)	4,542	274 (6.0)	1.53 (0.87,2.68)	
1 to <2	302	14 (4.6)	Ref	343	13 (3.8)	Ref	
2 to <4	293	17 (5.8)	1.29 (0.61,2.73)	316	16 (5.1)	1.25 (0.59,2.63)	
≥4	150	11 (7.3)	1.55 (0.64,3.73)	171	15 (8.8)	2.33 (1.09,4.98)	

BMI, body mass index; CI, confidence interval; HR, hazard ratio.

[a] Adjusted for age, sex, treatment group, body mass index, systolic blood pressure (SBP), smoking and drinking status, fasting glucose, total cholesterol, triglycerides, high-density lipoprotein cholesterol, total homocysteine, proteinuria, estimated glomerular filtration rate (eGFR) at baseline (the first measurement), as well as time-averaged SBP during treatment, if not stratified.

eTable 4. Sex-specific BMI status stratified analysis[a]

Subgroup	N	Events (%)	Adjusted HR (95% CI)	N	Events (%)	Adjusted HR (95% CI)	*P* for interaction
**Male**		**<24**			**≥24**		**0.878**
≤−5	151	11 (7.3)	1.47 (0.47–4.65)	222	26 (11.7)	2.73 (1.12–6.67)	
1 to −5	1,346	71 (5.3)	1.26 (0.46–3.46)	2–062	117 (5.7)	1.63 (0.71–3.74)	
1 to 2	96	4 (4.2)	Ref	173	7 (4.0)	Ref	
2 to 4	95	5 (5.3)	0.99 (0.25–3.98)	158	9 (5.7)	1.49 (0.52–4.30)	
≥4	46	4 (8.7)	2.07 (0.50–8.57)	73	10 (13.7)	3.43 (1.19–9.86)	
**Female**		**<24**			**≥24**		**0.669**
≤−5	194	17 (8.8)	1.22 (0.44–3.39)	462	52 (11.3)	2.58 (1.30–5.12)	
1 to −5	1,687	105 (6.2)	1.13 (0.46–2.79)	4–041	244 (6.0)	1.56 (0.83–2.96)	
1 to 2	98	5 (5.1)	Ref	278	11 (4.0)	Ref	
2 to 4	87	5 (5.7)	1.09 (0.32–3.78)	269	14 (5.2)	1.38 (0.61–3.12)	
≥4	53	3 (5.7)	1.11 (0.26–4.69)	149	9 (6.0)	1.57 (0.60–4.07)	

BMI, body mass index; CI, confidence interval; HR, hazard ratio.

[a] Adjusted for age, treatment group, systolic blood pressure (SBP), smoking and drinking status, fasting glucose, total cholesterol, triglycerides, high-density lipoprotein cholesterol, total homocysteine, proteinuria, estimated glomerular filtration rate (eGFR) at baseline (the first measurement), as well as time-averaged SBP during treatment.
